# Ginsenoside Rg1 attenuates the NASH phenotype by regulating the miR-375-3p/ATG2B/PTEN-AKT axis to mediate autophagy and pyroptosis

**DOI:** 10.1186/s12944-023-01787-2

**Published:** 2023-02-10

**Authors:** Xuanxin Chen, Wei Xue, Jia Zhang, Jiayi Peng, Wenxiang Huang

**Affiliations:** 1 Department of Infectious Diseases, The First Affiliated Hospital of Chongqing Medical University, Chongqing, 400016 China; 2 Department of Geriatrics, the First Affiliated Hospital of Chongqing Medical University, Chongqing, 400016 China

**Keywords:** NASH, Ginsenoside Rg1, miR-375-3p, ATG2B, Autophagy, Pyroptosis

## Abstract

**Background:**

Nonalcoholic steatohepatitis (NASH) is one of the most frequent liver diseases at present, and there is no radical treatment. The consequences of a variety of ginsenoside compounds on this situation have before been reported, however, the specific effect on the monomeric ginsenoside Rg1 (Rg1) and its associated underlying molecular mechanism stay unknown.

**Material and methods:**

In vitro, the cell models were constructed by exposing free fatty acids (FFAs) to HepG2 cells. A methionine and choline deficiency (MCD)-induced NASH mouse model was also established over 5–6 weeks of treatment. Rg1 is a traditional Chinese medicine monomer. These NASH models were treated with Rg1 and analyzed by qRT-PCR, Western Blot, sequencing, Oil red O staining, immunofluorescence, enzyme activity, HE staining, ELISA, double luciferase reporter assay, and immunohistochemistry.

**Results:**

Overexpression of ATG2B, an autophagy-related protein, attenuated lipid droplet accumulation and reduces ALT, AST, inflammatory cytokines, hydrogen peroxide, and pyroptosis in established mouse and cellular models of NASH and increased levels of ATP and autophagy. The binding sites of miR-375-3p and ATG2B were verified by bioinformatic prediction and a dual-luciferase reporter gene. Knockdown of miR-375-3p promoted autophagy and inhibited pyroptosis. ATG2B knockdown substantially attenuated the impact of miR-375-3p on NASH. Rg1 appears to regulate the occurrence and development of NASH inflammation through miR-375-3p and ATG2B in vitro and in vivo, and is regulated by PTEN-AKT pathway.

**Conclusions:**

This study showed that Rg1 participates in autophagy and pyroptosis through the miR-375-3p/ATG2B/PTEN-AKT pathway, thereby alleviating the occurrence and development of NASH, for that reason revealing Rg1 as a candidate drug for NASH.

**Supplementary Information:**

The online version contains supplementary material available at 10.1186/s12944-023-01787-2.

## Introduction

Nonalcoholic steatohepatitis (NASH) is a type of nonalcoholic fatty liver disorder (NAFLD), which includes hepatic steatosis, hepatocyte inflammation, and fibrosis, and can enhance into liver cirrhosis and even liver most cancers [1]. There is a lack of clinically fantastic pills to deal with the disease. Therefore, there is an urgent need to deeply understand the pathogenesis of NASH and find good therapeutic drugs to alleviate its occurrence and development.

Rg1 is a primary active ingredient, which is considered to be one of the most valuable herbs in traditional Chinese herbs such as ginseng. Rg1 has a robust impact on oxidative stress, reactive oxygen species, and anti-inflammatory processes, following studies [2–5]. In addition, it can additionally shield the liver, for example, it can minimize the capacity of TNF-α and different pro-inflammatory elements [6], and decrease the potential of liver fibrosis [7]. Rg1 has also been shown to improve liver function and inhibit apoptosis in mice with acute liver failure, indicating that autophagy is an essential protective function [8, 9]. The mechanism through which Rg1 regulates the incidence and development of NASH infection stays unclear.

Autophagy is a mobile self-degradation manner that degrades mobile proteins and broken or immoderate organelles by means of forming double-membrane autophagosomes [10, 11]. In latest years, extra and extra proof has proven that autophagy channels are blocked in the liver of NASH sufferers [12, 13]. In addition, deficiency of autophagy in hepatic sinusoidal endothelial cells in NASH sufferers has been proven to promote liver inflammation, endothelial-mesenchymal transformation, cell apoptosis, and the incidence and improvement of liver fibrosis in the early stage of NASH [14], suggesting that autophagy is carefully associated to liver disease. Autophagy-related genes (ATGs) are the primary regulators of the autophagy process, and their expression positively corresponds with physiological ranges of autophagy [15]. Endoplasmic reticulum stress (ERs) has been observed to result in apoptosis and autophagy, whilst ATGs modify the ensuing autophagy [16]. ATG2B is a member of the ATG family, and research have explored its function in numerous illnesses [17]. Nevertheless, the mechanism via which ATG2B influences autophagy and pyroptosis in NASH cells remains to be explored.

Pyroptosis is mainly dependent on the inflammasome, a new form of inflammatory cell death composed of pyrin domain 3 (NLRP3), ASC, and procaspase ase1 [18] of the NLR family. In the context of NASH, activation of NLRP3 inflammasome is triggered by lipotoxicity, organelle stress, and hepatocyte death, while exacerbates hepatic steatosis [19]. In addition, activation of NLRP3 inflammasome has additionally been proven to result in pyroptosis and promote the secretion of IL-1β [20]. A regulatory relationship between autophagy and pyroptosis has additionally been reported. For example, inhibition of autophagy induces the launch of LDH, activation of the NLRP3 inflammasome, and pyroptosis [21]. Liraglutide improves NASH via inhibiting the NLRP3 inflammasome and pyroptosis based totally on mitochondrial phagocytosis [22]. The learn about of the molecular mechanisms of autophagy and pyroptosis will assist similarly deepen our appreciation of the pyroptosis of NASH. In short, it is of fantastic importance for us to discover new treatment options for NASH.

MicroRNAs (miRNAs or miRs) play a key role in cancer and inflammation, but their role in NASH still needs to be explored. MiR-690 treatment has been reported to reduce fibrosis and steatosis in NASH [23] and restore specific kuffer cell function. Inhibition of miR-188-5p also significantly reduced HSC activation and proliferation via the PTEN/PI3K/AKT pathway, thereby inhibiting liver fibrosis [24]. It has been pronounced that miR-375-3p in OA can inhibit the expression of ATG2B in chondrocytes and inhibit autophagy, for that reason merchandising ERS [25].

In this study, we further explored whether Rg1 regulates the occurrence and development of NASH by regulating autophagy and pyroptosis through miRNA targeting ATG2B, providing a strong theoretical and experimental basis for new NASH therapies.

## Materials and methods

### Clinical specimens

From December 2020 to September 2022, liver specimens from six healthful controls (HC) and eight sufferers with NAFLD from the First Affiliated Hospital of Chongqing Medical University had been collected. Progress of liver injury Assessment of NASH based on different pathologies changes, increased fibrosis scores, ALT and AST levels (Table 1). Informed written consent of all NAFLD/NASH patients and healthy donors, and the protocol is certified by the First Affiliated Hospital of Chongqing Medical University, Chongqing, China (protocol code: 2022–048, March 2022).

**Table 1:** Clinical characteristics of human subject

	All	Healthy Control	NAFLD ( NAF = 2, NASH = 6)	*P*-value
	*n* = 14	*n* = 6	*n* = 8	
Age, years (mean ± S.D.)	49.07 ± 3.15	48.83 ± 3.13	49.25 ± 3.15	0.8241
Gender, male (n,%)	7 (50.00%)	2 (33.33%)	5 (62.50%)	0.5892
Serum ALT (U/l)	36.28 ± 15.10	19.15 ± 0.42	48.87 ± 5.39	0
Serum AST (U/l)	37.01 ± 14.79	20.90 ± 5.88	49.10 ± 3.98	0

Bold and italics numbers indicate significant differences as *P*-value < 0.05

*Abbreviations*: *NAFLD* Nonalcoholic fatty liver disease

### Cell culture and transfection

This literature shows that by establishing the NAFLD cell model by HepG2 cells [21], HepG2 cells have more obvious lipid accumulation than LO2 cells. HepG2 cells need to be cultured in DMEM medium containing 10% fetal bovine serum (Bioindustry, 1,707,254) and incubated at 5% CO2 at 37 ℃.

Briefly, 1 mM FFA solution was generated by mixing oleic and palmitic acids in a ratio of 2:1 and then dissolving the mixture in MEM supplemented with 1% bovine serum albumin (BSA) to enable the formation of a complex between FFA and BSA. Finally, 0.1% DMSO was boiled in a 55 ℃ water bath for 5 min and then run at a constant speed on a shaker for 12 h to dissolve. To establish a NASH cellular model, HepG2 cells were treated with 1 mM free fatty acids (FFAs) for 24 h.

In the FFA cell model, the concentration of Rg1 was 40 μmol/L. To block autophagy, 3-methyladenine (3-MA) and Rapa have been used for culture.MiR-375-3p used to be knocked down in vitro and cells had been transfected with mimics/inhibitors with the use of lipofectamine 3000 (Invitrogen, USA).

### Animals and treatments

NASH mice on an MCD eating regimen have been simulated in the use of 8-week-old male C57BL/6 mice. Study reported that the NASH mouse model [12] was constructed from mice fed the MCD diet to verify the changes in hepatic steatosis, inflammatory response, and fibrosis. MCD feed and standard feed were purchased from Nantong Trophy Feed Technology Company, MCD feed contains (per 1000 g): amino acid premix (methionine free) 175.7 g, methionine 0 g, choline chloride 0 g, sucrose 431.9 g, dextrin 50 g, corn starch 150.0 g, corn oil 100.0 g, cellulose 30.0 g, mineral mix 52.4 g. Mice were anesthetized with 1% pentobarbital sodium. The mice were killed and the eyes were quickly removed. Then, we collected the blood form the retroorbital vessels and stored at -80℃ for further study.

In a mouse animal model, gavage was performed with Rg1 30 mg / (kg**•**d) and serum and liver tissue were collected from each group after 4 weeks of continuous treatment. Then the mouse liver was resected and rinsed with normal saline. Part of the liver of every mouse was once reduced and constant in 4% paraformaldehyde answer and saved at room temperature for subsequent histological analysis.

### Hoechst staining

HepG2 cells (10^6^ cells /mL) were incubated with Hoechst 33,342 (5 μL) and PI (5 μL) (CA1120, Solarbio, Beijing, China) for 10 min. Then, fluorescence microscopy (Olympus) was used to observe the morphology of stained HepG2 cells. The test used to be repeated at least three times.

### Build the Rg1 target network

Bioinformatics databases (http://www.swisstargetprediction.ch/) have been used to predict Rg1 targets, and primarily based on the screening of 0.02 or greater threshold determination Rg1 key targets. Then, the protein–protein Interaction (PPI) community was once acquired on the database internet site (https://stringdb.org/).

### Argonaute 2 (AGO2) and RNA Immunoprecipitation (RIP)

MagnaRIP™ RNA-binding protein immunoprecipitation pack (Cat) precipitation and RIP lysis buffer (MA, USA) have been used, and blended with anti-Panago antibodies (MABE56, Microporous) and incubated with IgG antibody. Then, the Trizol reagent was added to collect RNA and protein from immunoprecipitation for subsequent analysis.

### Western blot

5 × 10^6^ HepG2 cells and 20 mg of mouse liver tissue were ground by sonication. After grinding on ice, add the appropriate volume of RIPA lysate and PMSF; the homogenizer will fully homogenate. Ultrasonic cell breaker over 3 times: 4℃ centrifuge 12 000 r/min for 15 min: leave supernatant: measure protein concentration by BCA, diluted with 5 protein loading buffer, fully denatured in boiling water for 8 min; protein sample followed by electrophoresis and membrane transfer to PVDF membrane; 5% nonfat milk powder closed for 1 h; 4℃ refrigerated chamber shaker overnight incubation corresponding primary antibody: TBST wash 3 for 5 min: secondary antibody on room temperature shaker for 1 h; TBST wash 3 for 5 min; ECL reagent development.

### QRT-PCR

Total RNA was isolated from HepG2 cells using RNAiso Plus (Takara, Kusatsu, Japan) and then sampled using the Fast SYBR Green PCR kit (Takara, Kyoto, Japan). The liver was removed from the mice, quickly washed quickly with ice saline, and one of the liver was stored in a frozen tube with RNAlater™ (Beyotime, Shanghai, China). The concentration and purity of RNA were measured with a spectrophotometer (NanoDrop 2000; Thermo Fisher). The cDNA was extracted from the ™RT kit (Takara, Kyoto, Japan). The inner manipulate corporations have been U6 and GAPDH. QRT-PCR was performed in three stages denaturation, annealing, and extension, and 40 cycles were performed. miRNA and mRNA expression degrees had been standardized in the usage of U6 or GAPDH. The primer sequence is proven in Table 2.

**Table 2:** Oligonucleotide sequences and RT-qPCR primers

Oligonucleotide sequences and RT-qPCR primers	
U6	GTGCTCGCTTCGGCAGCACATATACTAAAATTGGAACGATACAGAGAAGATTA GCATGGCCCCTGCGCAAGGATGACACGCAAATTCGTGAAGCGTTCCATATTTT
miR-375-3p	UUUGUUCGUUCGGCUCGCGUGA
ATG2B	Forward: 5’-AACTGCTGACGAATCCTCAGG-3’ Reverse: 5’-GGGGTTCCAGCTAGGTGAGA-3’
GAPDH	Forward: 5’-CTGGGCTACACTGAGCACC-3’ Reverse: 5’-AAGTGGTCGTTGAGGGCAATG-3’

### Enzyme-linked immunosorbent assays (ELISA)

Following the practice of the ELISA package (Cloud-Clone Corp. Wuhan, China), we employed the enzyme-linked immunosorbent assay to quantify the IL-6, TNF-α, and MCP-1 stages in the liver tissue and cells supernatants.

### Double luciferase reporter gene analysis

Wild-type and mutant sequences of ATG2B 3'-UTR had been developed and developed into luciferase receptor vectors and transfected into HepG2 cells for commentary via mimicking miR-375-3p. A twin luciferase assay package (Promega) used to be used to quantify luciferase undertaking after the normalization of enzyme activity.

### LDH release assay

LDH release in HepG2 cells have been decided on the usage of the LDH cytotoxicity assay package (Beyotime, C0016). Absorbance values (Thermo Fisher Scientific) have been examined at 490 nm with the use of an enzyme marker.

### Metabolic measurements and Triglyceride content detection

After the profitable institution of NASH mice and mobile models, we used Hitachi 7600 clinical analyzer to become aware of the content material of ALT and AST supernatant in mouse serum and phone supernatants. Intracellular ROS ranges had been detected with the aid of fluorescence microscopy. Take a look at the package (Jiancheng, China) used to be used to notice triglycerides (TG).

### HE and Oil red O staining

Mouse liver tissues have been constant with 4% paraformaldehyde, embedded in paraffin, sectioned, and histologically examined through HE staining. HepG2 cells were stained with an Oil red O (Sigma-Aldrich, USA) staining kit in the cells.

### Immunohistochemistry (IHC)

Tissue sections had been enclosed in PBS with 8% goat serum and incubated with an anti-NLRP3 antibody (Abcam, ab263899, diluted 1:1000). Sliced and cultured with Sheep Anti-Rabbit IgG (Abcam, ab6721, diluted 1:1000) at room temperature for 1 h. Finally, tissue sections had been stained with DAPI, immunolabeled and nuclear staining, and hematoxylin, and then found underneath a microscope.

### Analysis of immunofluorescence staining

HepG2 cells have been imfixed with 4% paraformaldehyde, first enclosed at room temperature with 5% BSA, and rabbit monoclonal antibody LC3 IgG (ab192890) was once used. The pattern used to be incubated with goat anti-Rabbit IgG (ab150077) for 1 h, and then the nucleus used to be stained with DAPI. Cells are considered thru a microscope.

### Flow cytometry

1 × 10^6^ HepG2 cells had been brought to acridine orange (Invitrogen, USA) for staining, and autophagy was once detected by way of glide cytometry. Then, 500 μl of the binding buffer used to be introduced to the phone microspheres and the phone answer used to be moved into microtubules protected with black paper. Then the cells have been Annexin 2 μl Annexin V-FITC/PI Double staining package (Invitrogen). Three biological repeats were performed.

### Transmission electron microscopy (TEM)

The HepG2 cells have been constant in 2.5% glutaraldehyde answer at 4 °C overnight. The cells had been then post-fixed in 1% citric acid solution, dehydrated in alcohol, and heated at 70 °C in a single day earlier than being organized for flat embedding in a LEICA EM UC7 ultrathin slicer and indicated through TEM.

### Statistical analysis

The above experiments had been repeated three times, all the use of mean ± SD. PRISM 7.0 (GraphPad, USA) software program for statistical analysis. Comparisons have assessed the use of students' T-tests. The records in group variations had been made the use of one-way evaluation of Student’s t-test. *P < 0.05, **P < 0.01, and ***P < 0.001.

## Results

### Bioinformatic analysis

**Fig. 1:**
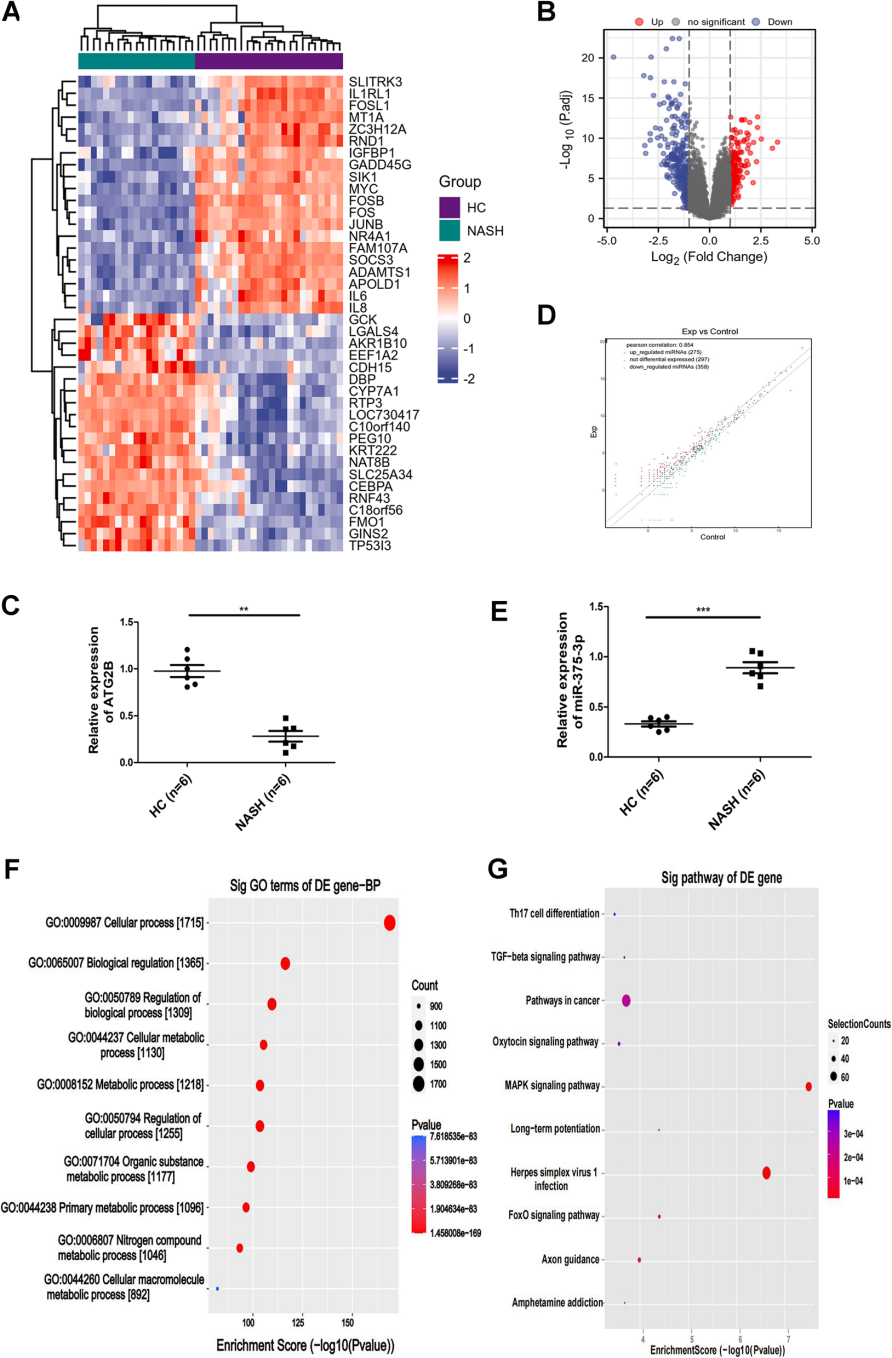
Differentially expressed miRNAs and mRNAs associated with autophagy in NASH. **A** Heatmap of pinnacle differentially expressed mRNA in NASH sufferers and preferred samples. The warmness map of the microarray records GSE89632. **B** Volcano plot displaying the upregulated (red) and downregulated (blue) genes in NASH sufferers (*P* < 0.05 and |Log2FC|≥ 1.5). **C** Relative expression of ATG2B in NASH sufferers and ordinary sufferers was analyzed by using qRT-PCR. **D** The scatter plot between two agencies for miRNA. **E** The relative expression of miRNA was detected by qRT-PCR. **F** GO enrichment evaluation of miRNA. **G** The KEGG signaling pathway of the miRNA. *P < 0.05, **P < 0.01, ***P < 0.001

The DESeq2 analysis carried out on the NASH and everyday donor (ND) samples with the use of the ArrayExpress database recognized differentially expressed mRNAs with fold modifications (FD) > 2 (log2fold > 1) (Fig. 1A). Volcano plot, displaying up-and down-regulation (red) genes in NASH patients (Fig. 1B). By qRT-PCR, a significant reduction in the ATG2B mRNA expression phase (*n* = 6) was observed in healthy patients (*n* = 8) (Fig. 1C). The values of the X and Y axes in the scatter plot use common CPM values, and the grey dots symbolize miRNAs except for differential expression (Fig. 1D). MiR-375-3p was detected employing qRT-PCR in sufferers with NASH in contrast with healthful donor samples (Fig. 1E). GO evaluation confirmed some integral organic methods for miRNAs enrichment (Fig. 1F). In addition, the signaling pathways associated with miRNA are shown (Fig. 1G).

### Effect of ATG2B expression in the FFA-induced NASH-like mobile mannequin

**Fig. 2:**
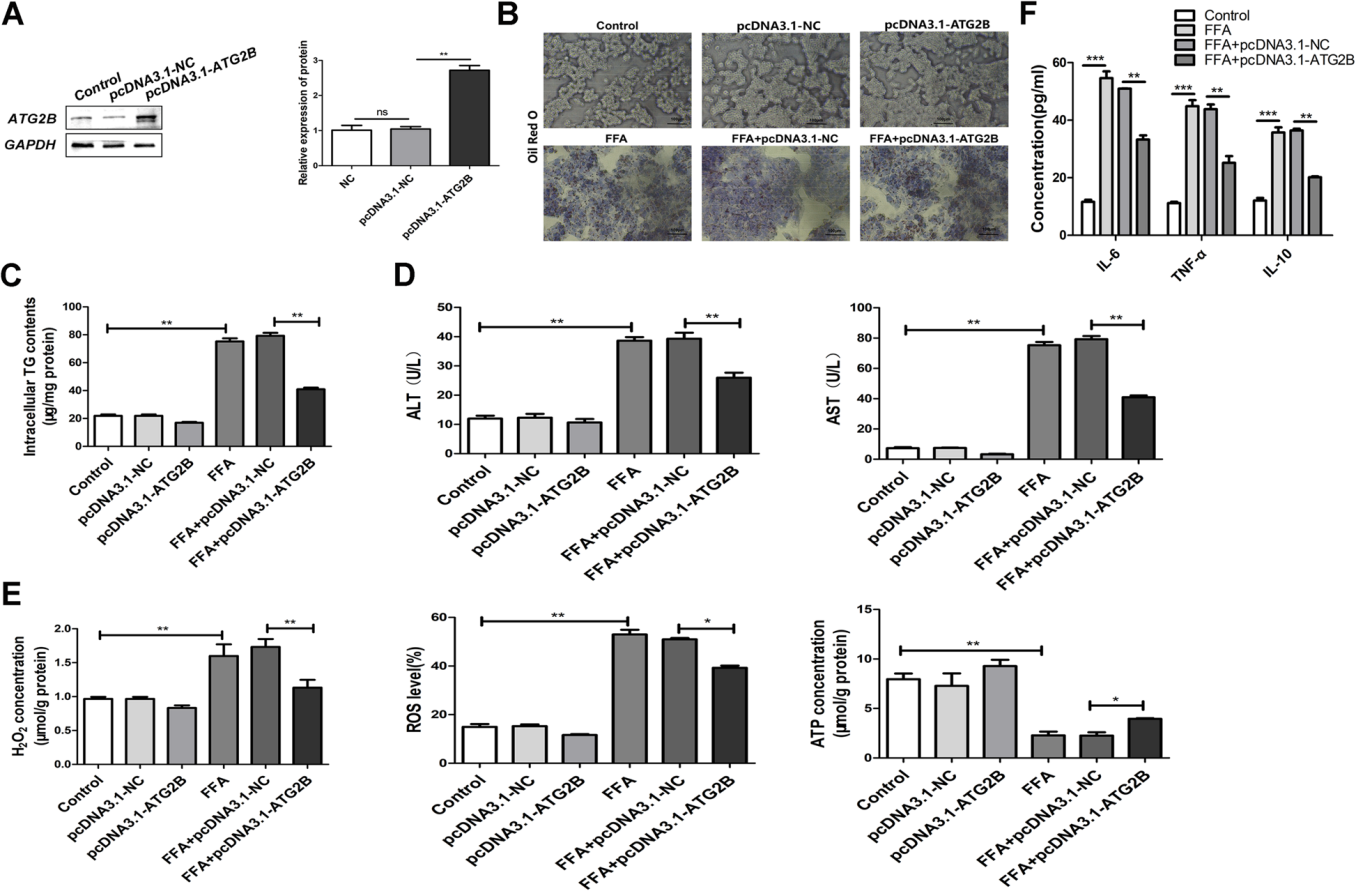
Role of ATG2B in NASH telephone model. **A** Relative mRNA stages of ATG2B as decided by using Western Blot and qRT-PCR. Error bars represent the SD. **B** HepG2 cells had been transfected with pcDNA3.1-NC or ATG2B and then dealt with or besides FFA (1 mM) for forty-eight hours. Representative pics exhibit the handled HepG2 cells (scale bar: four hundred μm) and Oil Red O staining. **C** TG ranges in traditional supernatants. **D** ALT and AST range in way of life supernatants. **E** H_2_O_2_ concentration, ROS level, and ATP attention in HepG2 cells. **F** The expressions of IL-6, TNF-α, and IL-10 were detected by ELISA. *P < 0.05, **P < 0.01, ***P < 0.001

HepG2 cells have been chosen to set up the NASH-like telephone model. The expression of ATG2B can be decided by using Western blotting. GAPDH was once used as the loading manage (Fig. 2A). The FFA-treated group significantly improved its accumulation of lipid droplets, while transfection of pcDNA3.1-ATG2B significantly reduced this accumulation (Fig. 2B). As proven in Fig. 2C-F, the degrees of TG, ALT, AST, H_2_O_2_, ROS, IL-6, TNF-α, and IL-10 had been appreciably increased, whilst the ATP degree used to be appreciably reduced in the FFA-induced NASH cell-like model.

### ATG2B inhibits hepatocyte pyroptosis in an autophagy-dependent manner caused by FFA

**Fig. 3:**
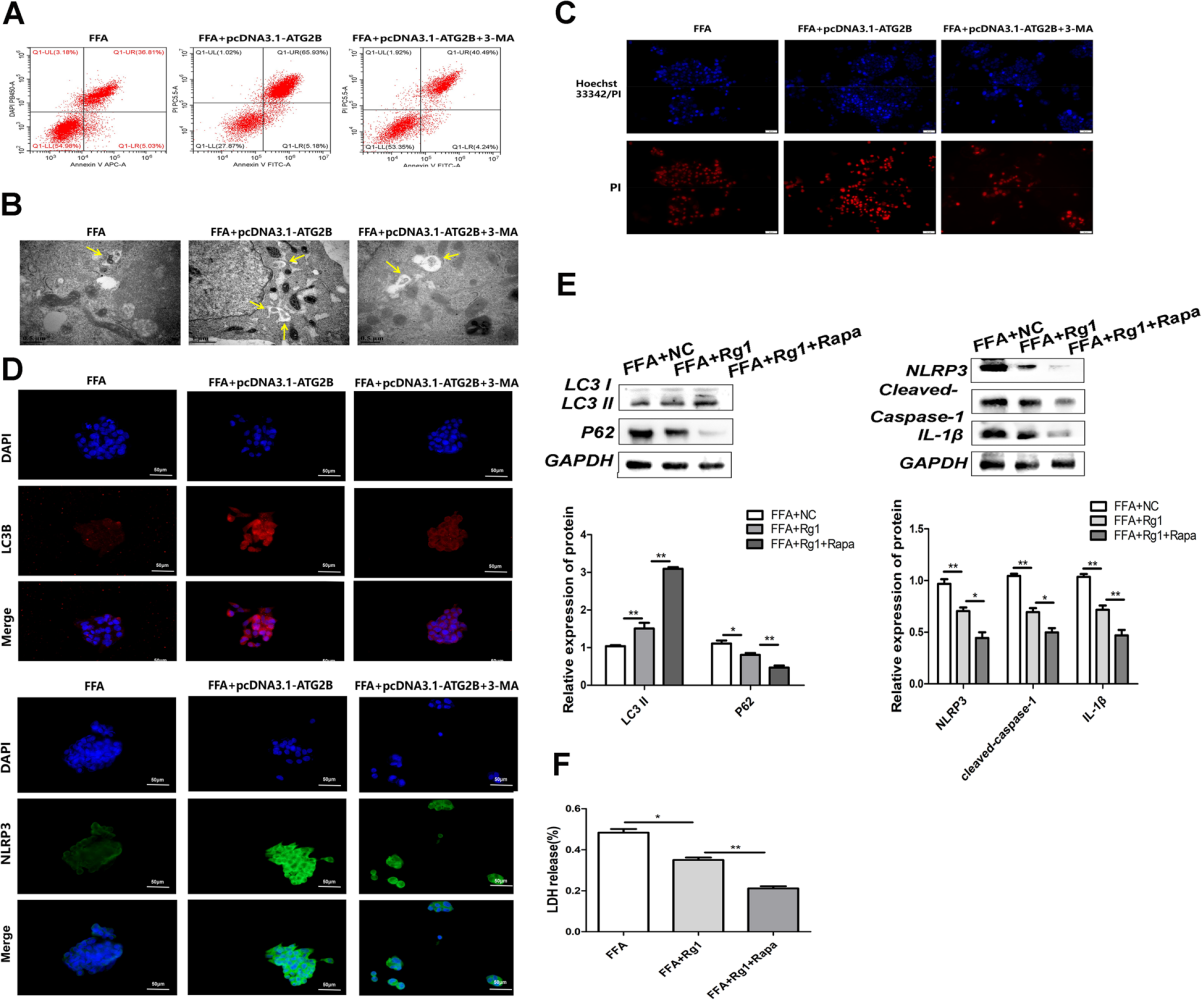
ATG2B regulates autophagy and pyroptosis in NASH models. **A** HepG2 telephone brought on by way of FFA apoptosis as detected through going with the flow cytometry assay. **B** Representative TEM photo (scale bar: 800 nm) of autophagosomes (indicated with the aid of purple arrows) in the NASH. **C** HepG2 cells are stained pink or vibrant red by way of hoechst 33,342/PI double staining (magnification, × 400) and the nucleus is no longer stained. Arrows point out PI-positive cells. Scale bar = 20 μm. **D** Immunofluorescence picture displaying LC3 and NLRP3 expression (red) in HepG2 cells brought about through FFA. **E** Western Blot showed autophagy and pyroptosis in the Rg1 and Rg1 + Rapa groups. **F** Release of LDH. *P < 0.05, **P < 0.01, ***P < 0.001

ATG2B-induced apoptosis was once indicated via glide cytometry, whereas the addition of 3-MA reversed the apoptosis (Fig. 3A). Transmission electron microscopy (TEM) confirmed that the variety of autophagosomes in pcDNA3.1-ATG2B increased, however, this was once ameliorated with the addition of 3-MA (Fig. 3B). Hoechst 33,342/PI double staining confirmed that the proportion of PI-positive HepG2 cells transfected with pcDNA3.1-ATG2B used to be greater than that in the control group, and this proportion was once down-regulated after 3-MA remedy (Fig. 3C). In vitro, the ranges of the autophagy markers LC3 and NLRP3 have been proven in the liver of NASH cells, whilst 3-MA reversed the impact of pcDNA3.1-ATG2B (Fig. 3D). Rg1 group promoted autophagy and inhibited pyroptosis, and the addition of Rapamycin (Rapa) intensified the promotion of autophagy and inhibited pyroptosis reaction (Fig. 3E). Similarly, Rg1 group inhibited NASH-induced LDH release, while Rg1 + Rapa increased LDH release (Fig. 3F).

### MiR-375-3p targets ATG2B in NASH cells

**Fig. 4:**
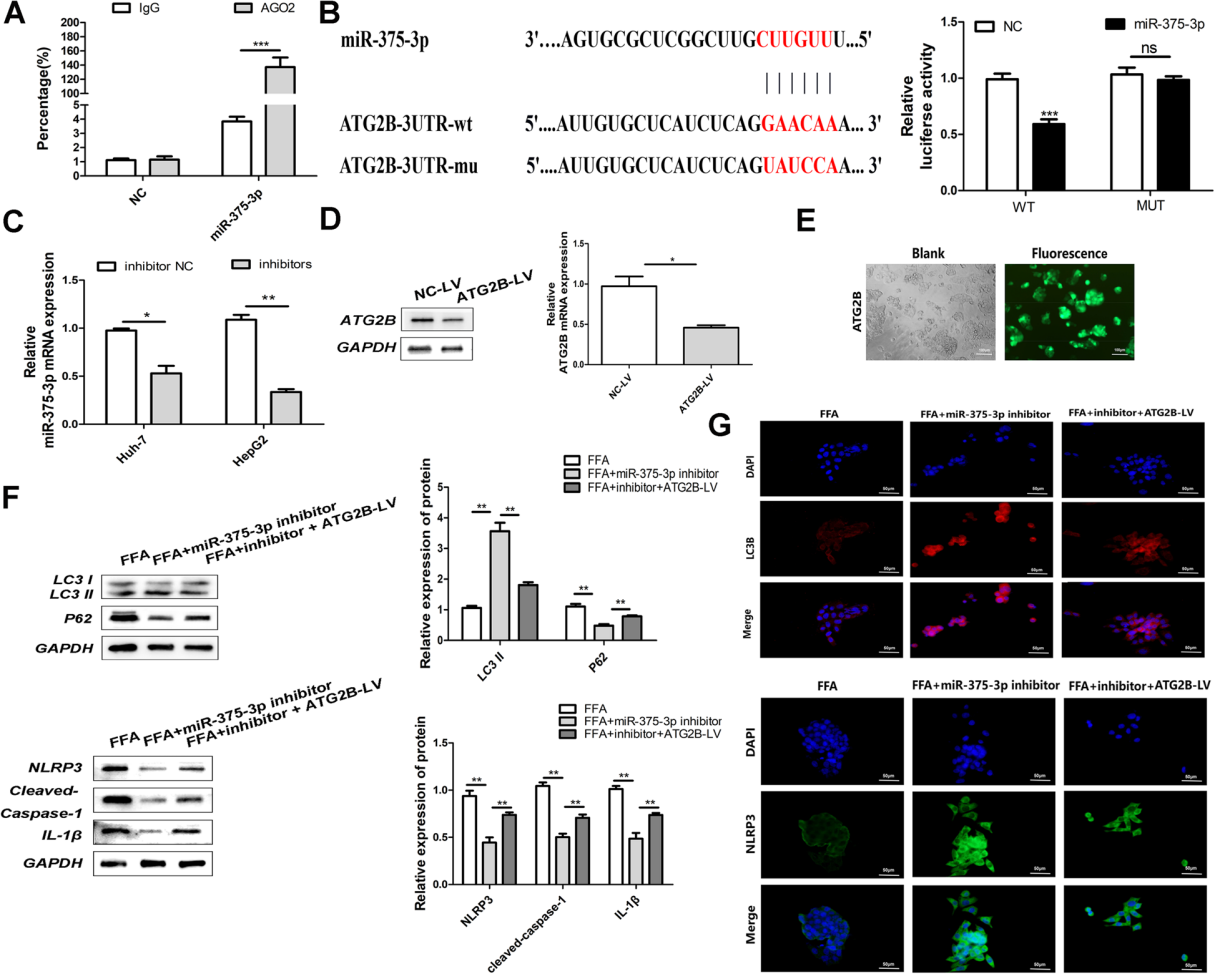
Interaction of miR-375-3p with ATG2B. **A** The binding of miR-375-3p to AGO2 was verified by AGO2-RIP experiment. **B** Data from bioinformatics (TargetScan) confirmed the combination. **C** Effect of transfection of the miR-375-3p inhibitor in HepG2 and Huh-7 cells. **D** The protein expression after transfection of ATG2B-LV was detected by Western Blot. **E** Green fluorescent protein to point out transfection effectivity in HepG2 cells triggered by using FFA. **F** Western Blot detection of LC3, P62, NLRP3, cleaved-caspase-1, and IL-1β protein expression. **G** LC3 and NLRP3 expression as detected via fluorescence microscopy. *P < 0.05, **P < 0.01, ***P < 0.001

The AGO2-RIP scan was once performed in HepG2 cells. After the AGO2 pull-down, AGO2-3p was once drastically enriched, indicating the interplay between AGO2 and miR-375-3p (Fig. 4A). The action mechanism of miR-375-3p was analyzed by TargetScan bioinformatics study. ATG2B used to be validated as a conceivable goal of miR-375-3p with the aid of outcomes got from the twin luciferase assay (Fig. 4B). Compared with the NC group, miR-375-3p inhibitors increased the expression of ATG2B (Fig. 4C). ATG2B overexpression used to be tested via Western Blot (Fig. 4D). Fluorescence microscopy confirmed the transfection efficiency of ATG2B (Fig. 4E). In addition, effects of the Western blot confirmed that LC3 was once upregulated whilst p62, NLRP3, cleaved-caspase-1, and IL-1β had been downregulated in the inhibitor group. In contrast, the transfection of the miR-375-3p inhibitor + ATG2B-LV produced the contrary effect, with cells exhibiting a decreased percentage of LC3 and greater expression stages of p62, NLRP3, cleaved-caspase-1, and IL-1β (Fig. 4F). In vitro evaluation additionally printed that the expression of LC3 and NRLP3 as decided by using immunofluorescence used to be altered utilizing the miR-375-3p inhibitor, whereas ATG2B-LV restored the expression of LC3 and NRLP3 proteins in the FFA-induced NASH cell-like model (Fig. 4G).

### Rg1 regulates autophagy and pyroptosis in fatty liver in vivo

**Fig. 5:**
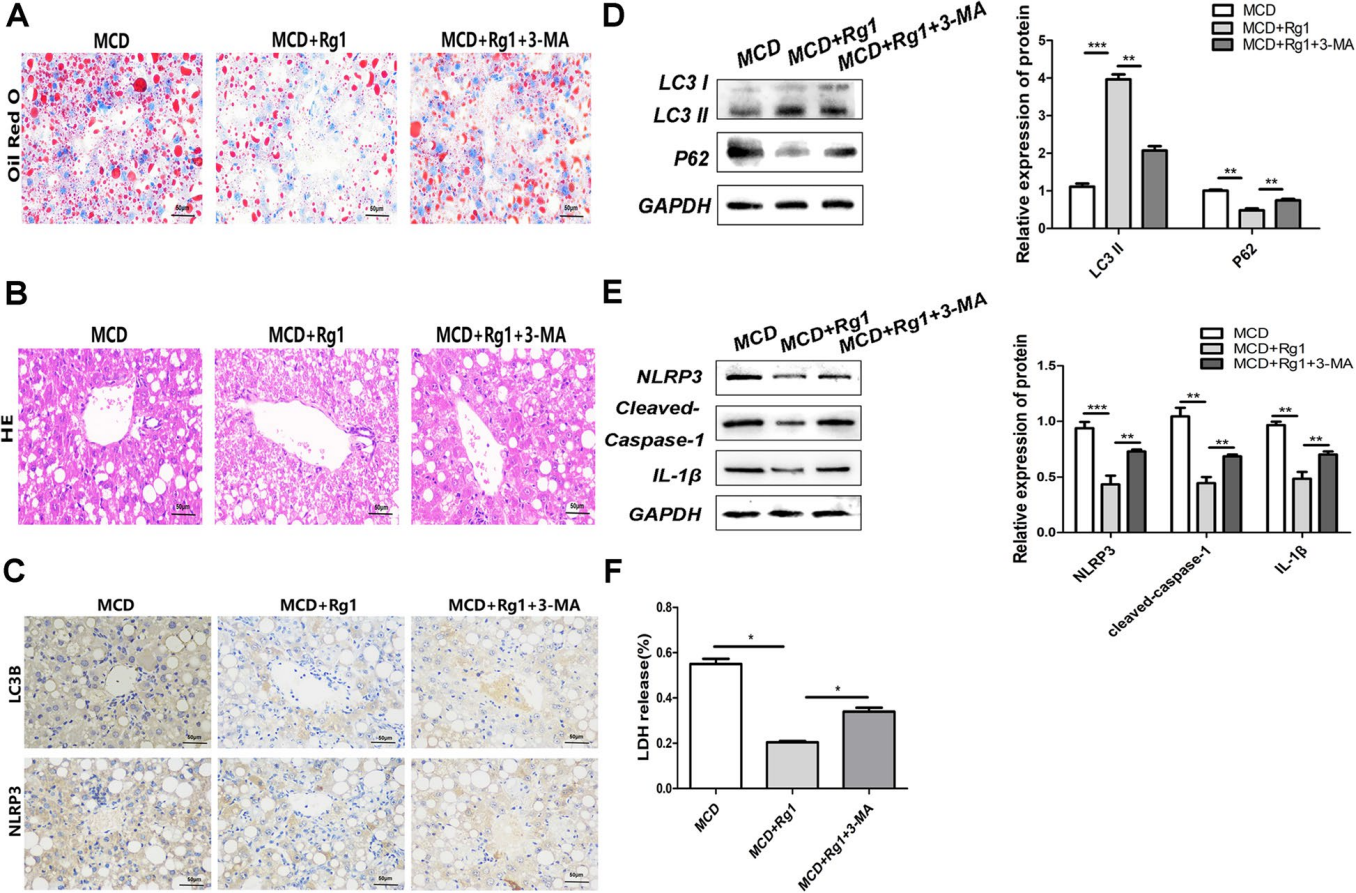
Rg1 improves fatty liver in vivo. **A** Oil red O staining. **B** Representative photomicrographs of HE staining. **C** Validation of LC3B and NLRP3 protein expression through the usage of immunohistochemical staining. **D-E** Western Blot detection of the protein expression of LC3, P62, ATG2B, NLRP3, cleaved-caspase-1, and IL-1β in the NC, MCD + Rg1, and MCD + Rg1 + 3-MA groups. GAPDH was once used as the loading manipulate **F** Release of LDH. *P < 0.05, **P < 0.01, ***P < 0.001

Oil red O (Fig. 5A) and HE (Fig. 5B) staining confirmed that in contrast with the management group, the liver tissue harm in the HFD-treated MCD + Rg1 + 3-MA group was once greater full-size than that in the MCD + Rg1 group, with observable fats vesicles and multiplied lipid droplets. Immunohistochemistry (Fig. 5C) showed that the enlargement in LC3B and NLRP3 in the MCD + Rg1 group used to be reversed by using the autophagy inhibitor 3-MA. It used to be established using Western blotting that in contrast with the MCD + Rg1 group, the MCD + Rg1 + 3-MA group had downregulated LC3 alongside expanded P62, NLRP3, cleaved-caspase-1, and IL-1β (Fig. 5D-E). The MCD + Rg1 group exhibited an amplify in the NASH-induced launch of LDH, whilst 3-MA restored the LDH launch to everyday stages (Fig. 5F).

### Validation of the PTEN/AKT pathway in vitro and in vivo

**Fig. 6:**
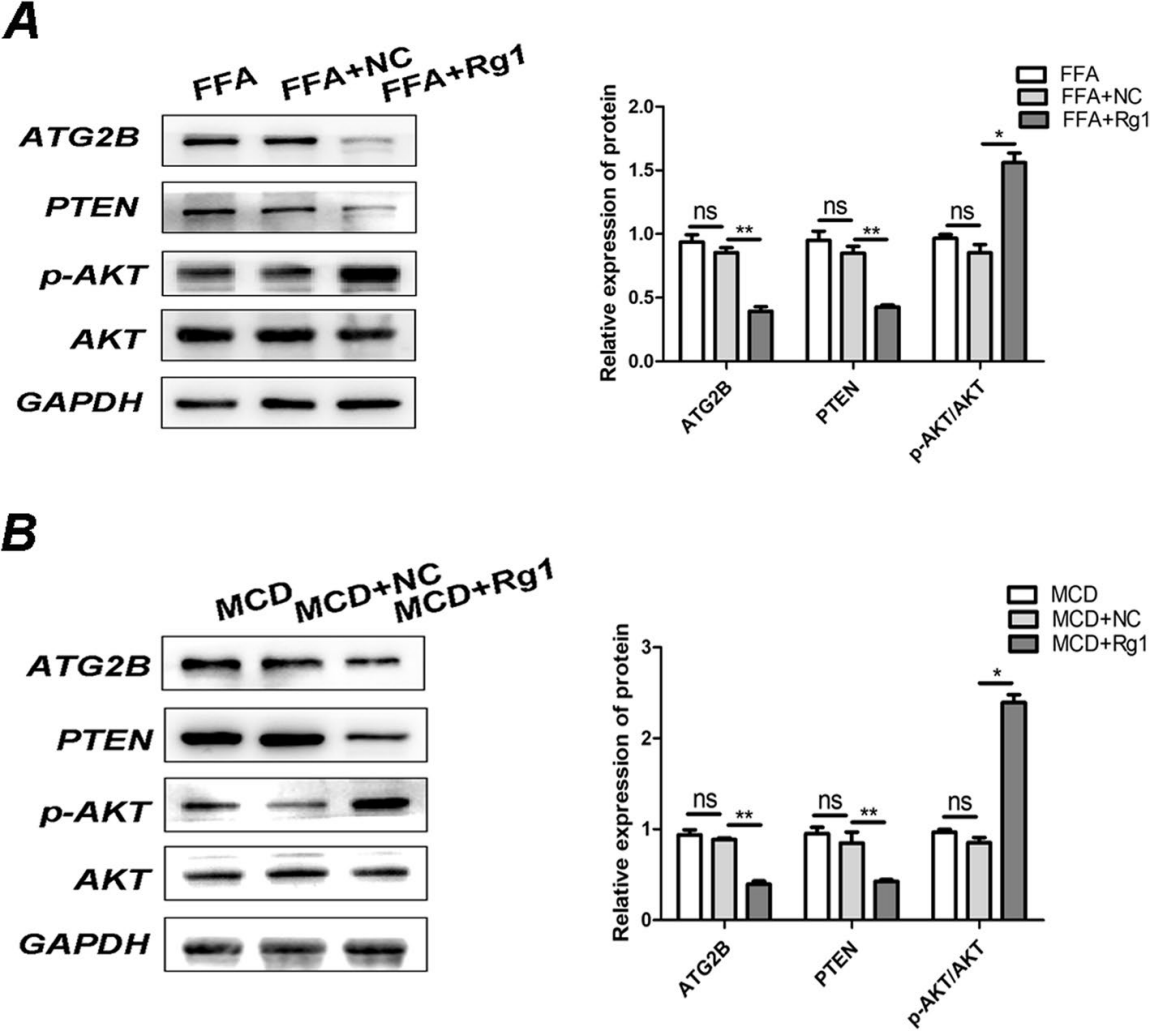
To look at the mechanism of the PTEN/AKT pathway. **A-B** Western blot evaluation of ATG2B, PTEN, p-AKT, and AKT in the NASH models. *P < 0.05, **P < 0.01, ***P < 0.001

In the FFA cell model, ATG2B used to be knocked down, the expression of PTEN used to be downregulated, and the p-AKT/AKT ratio used to be improved in the FFA + Rg1 group in contrast with the FFA group (Fig. 6A). In animal models, the expression of ATG2B and PTEN after transfection with Rg1 was down-regulated and the p-AKT/AKT ratio was up-regulated compared with the MCD group (Fig. 6B).

## Discussion

Nonalcoholic steatohepatitis (NASH) is a continual liver sickness brought on through more than one element for which there is presently no radical drug treatment. The pathogenesis of NASH stays underexplored [26]. NASH is on the whole characterized by immoderate fat deposition in the liver and the dysregulation of lipid metabolism and reactive oxygen species [27, 28]. At present, we comprehend that dietary recommendations and bodily workouts are the major techniques of NASH treatment [29], however, we need to discover new objectives from the pathogenesis of NASH, to suggest greater high-quality therapy methods. Compounds extracted from herbal merchandise are increasingly being used to deal with NASH due to the fact of their excessive efficacy and minimal facet consequences [30]. Although ginseng has been used in the normal cure of a variety of illnesses for extra than 2000 years, its actual medical fee and modern-day drug utilization cost have now not been in reality mirrored [31]. Recent research on Rg1 has established that it is an essential bioactive issue in Panax ginseng. It is of high-quality medical value to discover the achievable drug fee of Rg1 [32]. Up to now, the regulatory impact of Rg1 focused on ATG2B on NASH has no longer been reported. In our study, Rg1 focused on ATG2B drastically inhibited lipid droplet formation in steatosis HepG2 cells, suggesting that Rg1 should be a doable therapeutic agent for the remedy of NASH. Much research have proven that autophagy and hepatic lipid metabolism are interrelated [33]. It has been stated that autophagy can efficaciously stop the improvement of hepatic steatosis via mediating lipid metabolism to minimize triglycerides [34]. In addition, inhibition of autophagy can set off oxidative stress and speed up the activation of the NLRP3 inflammasome, main to pyroptosis [35].

Ginseng enjoys the reputation of "King of Chinese Herbal medicine" in traditional Chinese medicine. Although ginseng has been traditionally used to treat diseases for more than 2,000 years, people's understanding of Chinese herbal medicine is still skewed. Ginsenoside is an important bioactive component of ginseng. As a monomer, its mechanism needs to be further explored to provide scientific support for the traditional uses of ginseng. Therefore, the potential application value of Rg1 can be further studied, and the discovery of basic TCM drugs can be greatly promoted.

In addition, in NASH model, Rg1 can promote autophagy, inhibit pyroptosis, and restrict the expression of inflammatory factors in addition to alleviating lipid deposition. Therefore, we hypothesize that Rg1 may alter autophagy and pyroptosis and thus NASH. Similarly, autophagy inhibitors inhibited the inhibition of lipid droplet formation via Rg1, more suitable for the expression of pyroptosis-related proteins, and improved the launch of LDH. Rg1 improves liver failure by using regulating autophagy, ATG2B, and the PTEN/AKT pathway to suppress inflammation. This mixed proof helps our speculation that Rg1 ameliorates NASH via regulating autophagy and pyroptosis in hepatocytes.

There is proof that miRNAs play a key regulatory function in NASH [36]. Potential aims for differentially expressed mirnas are concept to play roles in lipid metabolism, apoptosis, and inflammation. For example, overexpression of miR-142-5p inhibits the improvement of NASH by using the JAK-STAT signaling pathway. miR-142-5p may additionally additionally be a new goal for NASH remedy [37]. In addition, miR-223 ameliorates NASH via inflammatory genes in hepatocytes [38]. miR-296 modifies fats apoptosis by means of finding p53 regulated apoptosis [39]. All these guides our experimental speculation that miR-375-3p can modify the prevalence and improvement of NASH.

Nonalcoholic fatty liver disorder development is accelerated by using the splenic law of liver PTEN/AKT [40]. Therefore, we conjectured the impact of the PTEN/AKT pathway on NASH in our experiment. This study also confirmed that Rg1 reduced the changes in PTEN and p-AKT/AKT ratios in NASH cells and animal models, and confirmed that Rg1 treated NASH through the PTEN/AKT pathway in vivo and in vitro.

### Comparisons with other studies and what does the current work add to the existing knowledge

Available data suggest that Rg1 is used to treat lipid degeneration and inflammation in NASH cells and animal models. However, there have been no clinical studies of Rg1. Including the current findings that a) Rg1 is a monomer of ginsenoside; b) After Rg1 treatment, NASH cells and mouse symptoms were improved through the miR-375-3p/ATG2B/PTEN-ATKT signaling pathway. These new findings extend previous findings and support the idea that Rg1 can improve the development of NASH.

### Study strengths and limitations

The advantage of this study is that Rg1 is a monomer for NASH therapy, and the results also suggest that Rg1 can improve the progression of NASH in vitro via miR-375-3p/ATG2B/PTEN-AKT. The study has several limitations. First of all, Rg1 belongs to a monomer, and multiple monomer combinations are needed to further study the therapeutic progress of NASH. Secondly, whether the therapeutic effect of Rg1 in humans is affected by miR-375-3p/ATG2B/PTEN-AKT needs to be further determined.

## Conclusion

In conclusion, the present work suggests that Rg1 can act as a liver protector for NASH. This study demonstrated for the first time that Rg1 promotes autophagy, suppresses pyroptosis, and alleviates the occurrence of NASH through the miR-375-3p / ATG2B / PTEN-AKT pathway. Our data provide novel insights into the targeting pathways involved in NASH and highlight a new class of therapies for naturally derived compounds that have not been well studied. Therefore, it is hoped that Rg1 can be applied in clinical studies to provide new ideas for the application of Rg1 to the clinical therapeutic target of NASH.

## Supplementary Information

**Additional file 1. **

**Additional file 2. **

**Additional file 3. **

**Additional file 4. **

**Additional file 5. **

## Data Availability

All data generated or analyzed in this study are available from the corresponding author for reasonable request.

## Abbreviations

NASH: Nonalcoholic steatohepatitis
NAFLD: Nonalcoholic fatty liver disorder
ATGs: Autophagy-related genes
FFAs: Free fatty acids
MCD: Methionine and choline deficiency
ERS: Endoplasmic reticulum stress
3-MA: 3-Methyladenine
PPI: Protein–protein Interaction
ALT: Alanine aminotransferase
AST: Aspartate aminotransferase
qRT-PCR: Quantitative real-time polymerase chain reaction
TC: Total cholesterol
TG: Triglyceride
