# Supplementary material for: Ginsenoside Rg1 attenuates the NASH phenotype by regulating the miR-375-3p/ATG2B/PTEN-AKT axis to mediate autophagy and pyroptosis
Source: Lipids Health Dis. 2023 Feb 10;22:22. doi: 10.1186/s12944-023-01787-2 (PMC9912620; doi:10.1186/s12944-023-01787-2)

Fig.2A

ATG2B


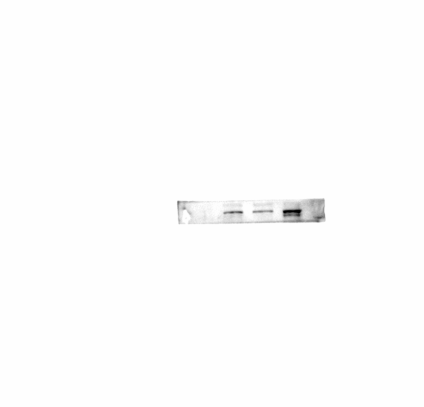


GAPDH


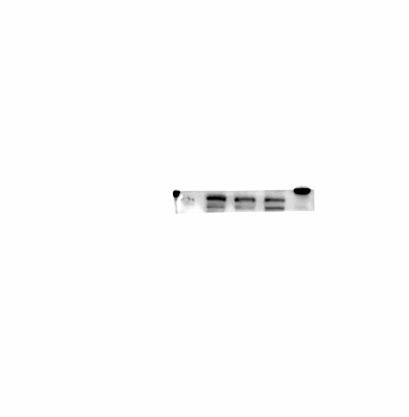


Fig.3E

LC3


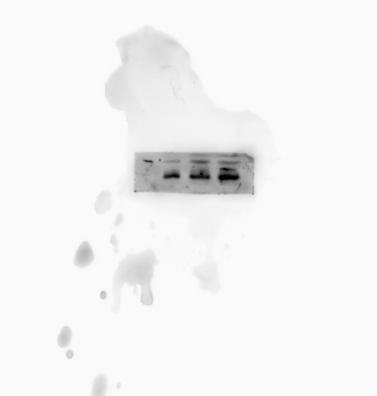


P62





GAPDH





NLRP3





cleaved-caspase-1


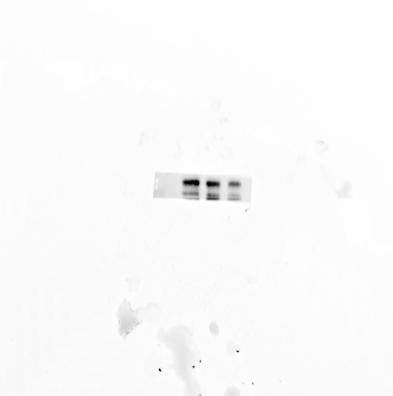


IL-1β


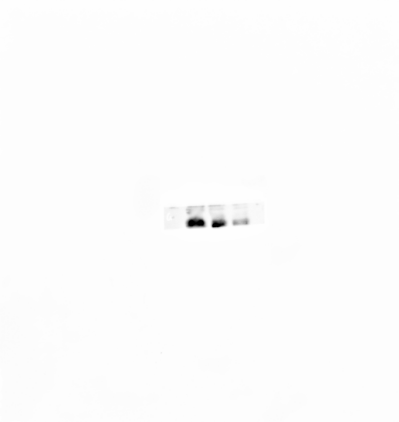


GAPDH


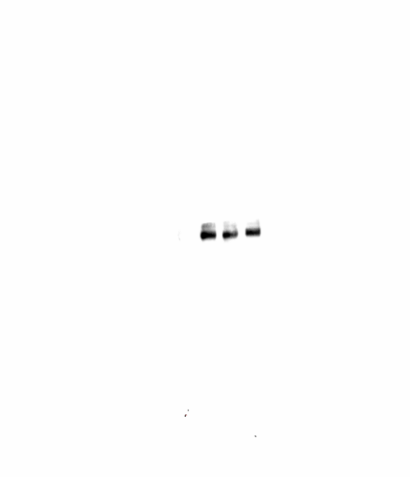


Fig.4D

ATG2B


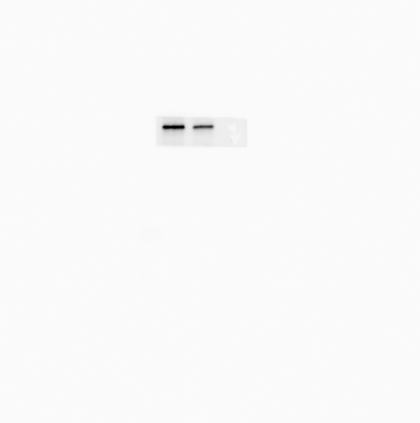


GAPDH


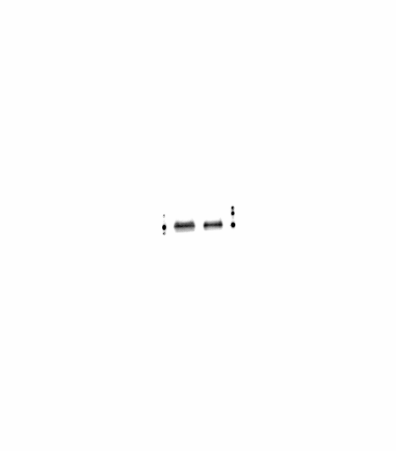


Fig.4F

LC3


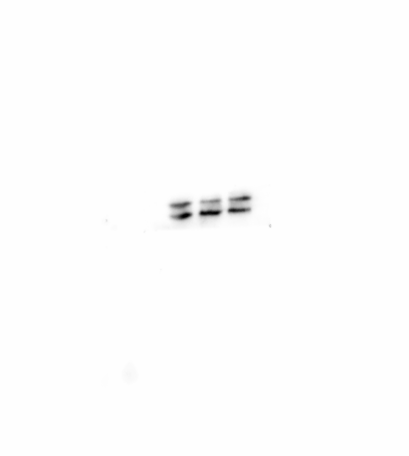


P62


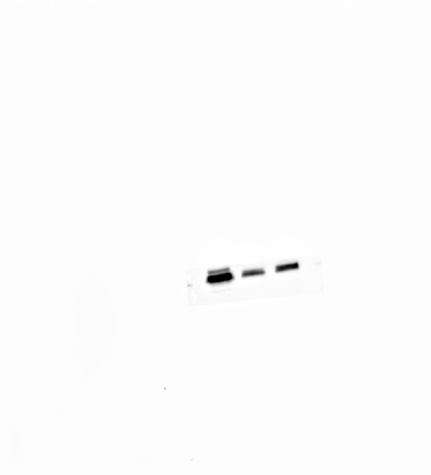


GAPDH


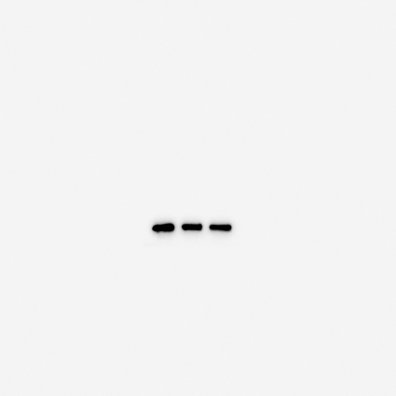


NLRP3


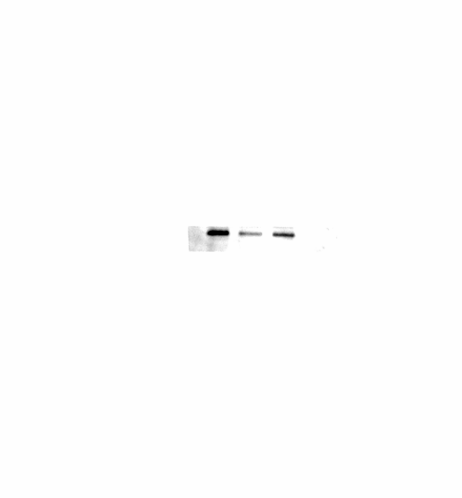


cleaved-caspase-1


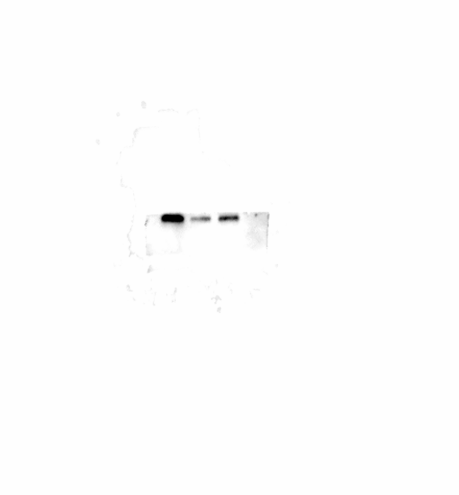


IL-1β


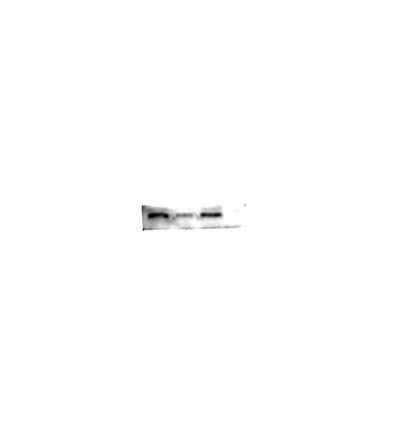


GAPDH


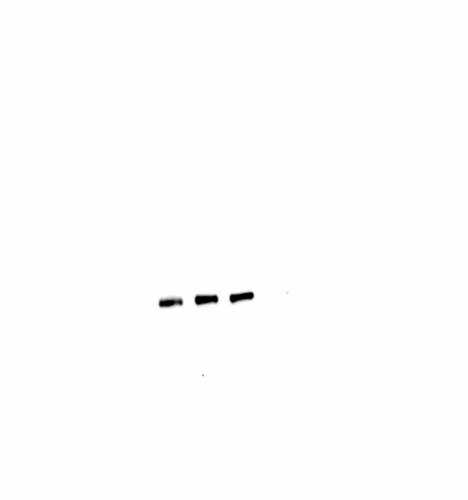


Fig.5D

LC3


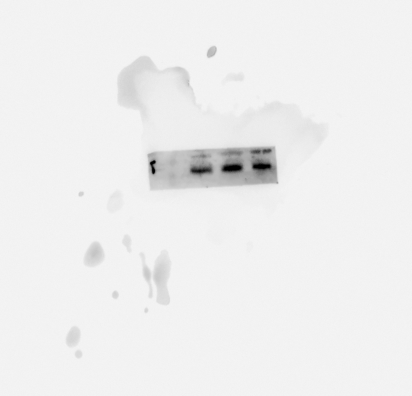


P62


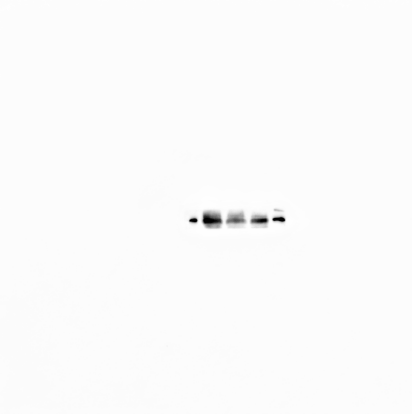


GAPDH


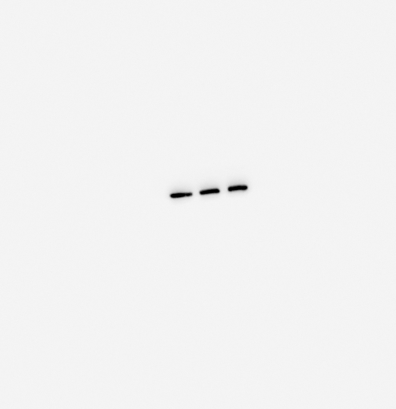


Fig.5D

NLRP3


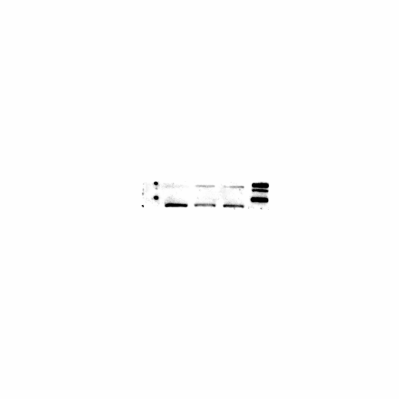


cleaved-caspase-1


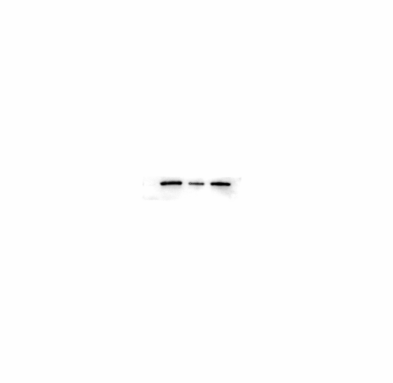


IL-1β


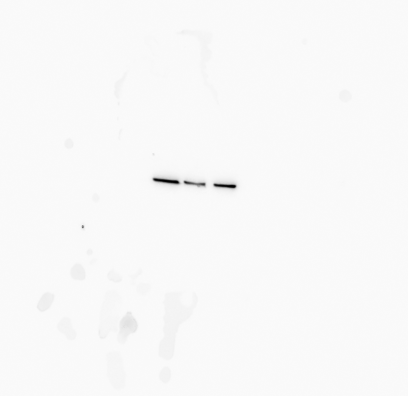


GAPDH


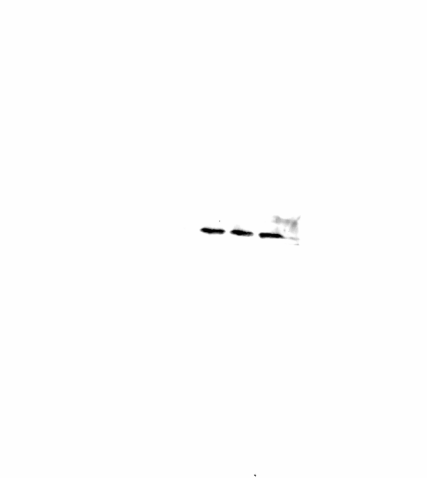


Fig.6A

ATG2B


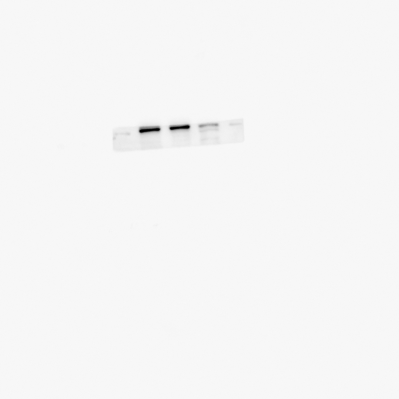


PTEN


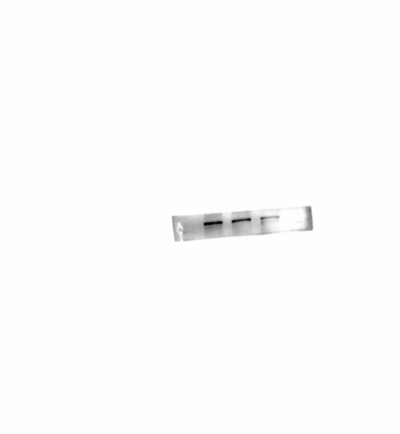


p-AKT


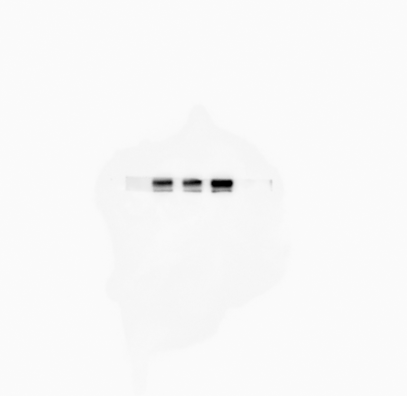


AKT





GAPDH


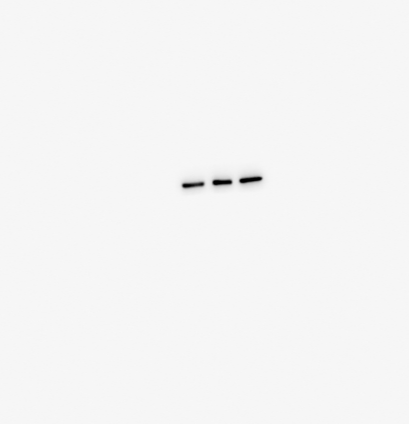


Fig.6B

ATG2B


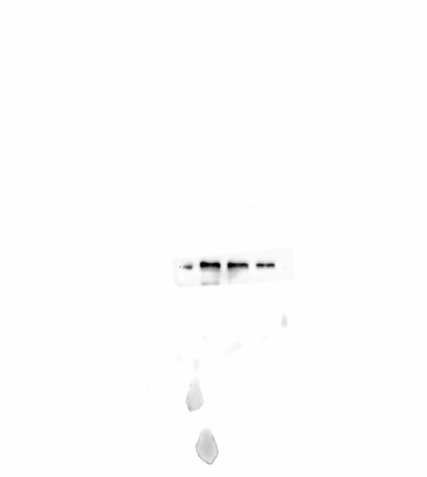


PTEN


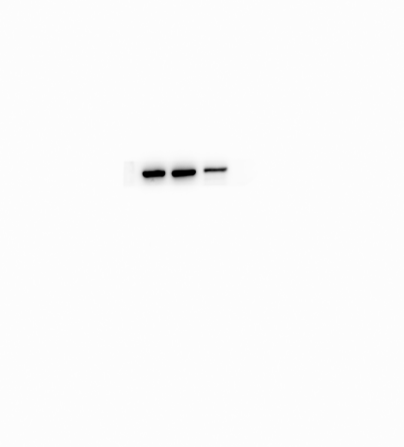


p-AKT


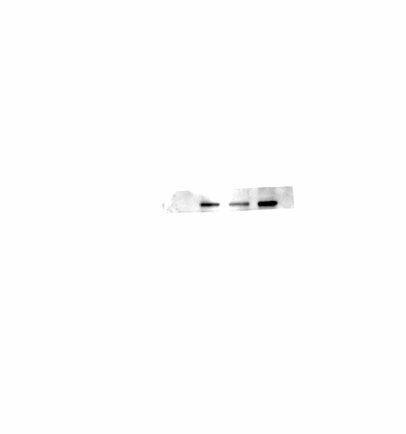


AKT


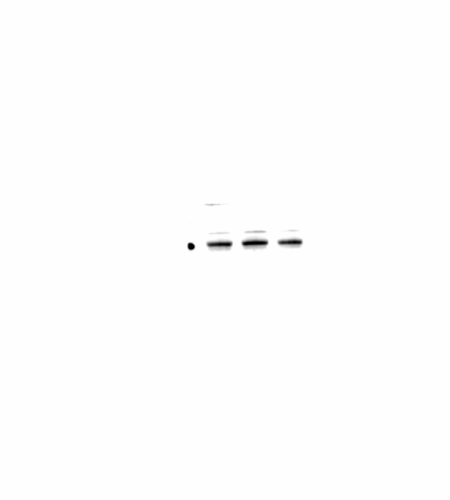


GAPDH


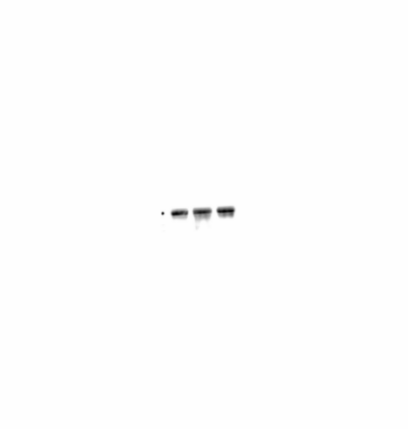

Supplement: Supplementary file 2 — Additional file 2. [file 12944_2023_1787_MOESM2_ESM.docx]
