# Supplementary material for: Ginsenoside Rg1 attenuates the NASH phenotype by regulating the miR-375-3p/ATG2B/PTEN-AKT axis to mediate autophagy and pyroptosis
Source: Lipids Health Dis. 2023 Feb 10;22:22. doi: 10.1186/s12944-023-01787-2 (PMC9912620; doi:10.1186/s12944-023-01787-2)
Supplement: Supplementary file 3 — Additional file 3. [file 12944_2023_1787_MOESM3_ESM.pdf]

重庆医科大学附属第一医院伦理委员会

审批意见书

申请人:黄文祥

申请审批项目名称:miRNA 通过 ATG2B 调节非酒精性脂肪肝病作用及机制研究

审查日期:2022 年 03 月 23 日

批准日期:2022 年 03 月 23 日

审查批号:2022 年科研伦理（2022-048）

经伦理委员会审查，本项目所提交的研究方案及知情同意书等资料符合医学伦理原则和赫尔辛基宣言的各项要求，研究设计具有科学根据，没有给受试者带来不必要的危险，对受试者的安全和隐私给予了最大限度的保护，批准该项目按预定方案实施。

重庆医科大学附属第一医院伦理委员会

2022 年 03 月 23 日

重庆医科大学附属第一医院伦理委员会
