# Supplementary material for: Ginsenoside Rg1 attenuates the NASH phenotype by regulating the miR-375-3p/ATG2B/PTEN-AKT axis to mediate autophagy and pyroptosis
Source: Lipids Health Dis. 2023 Feb 10;22:22. doi: 10.1186/s12944-023-01787-2 (PMC9912620; doi:10.1186/s12944-023-01787-2)
Supplement: Supplementary file 4 — Additional file 4. [file 12944_2023_1787_MOESM4_ESM.pdf]

EditorBar
北京艾迪润色

EditorBar Language Editing
No.35, Tsinghua East Road, Beijing, China 100083
Email: runse@editorbar.com Phone:+86-10-5620-8614

CERTIFICATE OF LANGUAGE EDITING

The English writing of the following manuscript was carefully edited by a native English speaker.

Manuscript Information

ID	AE202209100363
Editing date	2022-12-20
Title	Ginsenoside Rg1 attenuates the NASH phenotype by regulating the miR-375-3p/ATG2B/PTEN-AKT axis to mediate autophagy and pyroptosis
Corresponding author	Wenxiang Huang
Language writing before editing	☐ Very poor ☐ Poor ☐ Fair ☐ Good ☐ Very good ☑ Excellent
Recommendation after language editing	☐ Submitting to target journal directly<br>☑ Submitting to target journal after minor revision<br>☐ Re-editing required after major revision<br>☐ Not suitable for publication
Overview comments	The paper was written extremely well, in near-fluent English. There are many opportunities for revision, and several instances of missing text that the author can fill. There are also opportunities to clarify the text for the readers, and these have been indicated throughout the document. I wish you all the best.

Edited by

Tatyana L.

Senior Editor
The University of Sydney
Language Editing

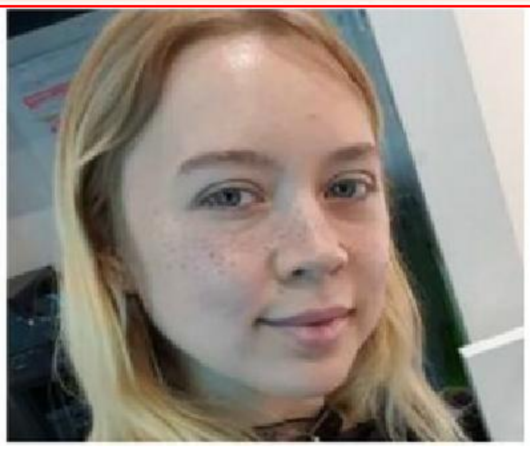

Certificate Issued by

Dr. Jason Qee

Editor in Chief
Editorbar Language Editing, Beijing, China
runse@editorbar.comwww.editorbar.com

Certificate link:www.editorbar.com/order/cert/AE202209100363
